# Calcium Silicate Improved Bioactivity and Mechanical Properties of Poly(3-hydroxybutyrate-co-3-hydroxyvalerate) Scaffolds

**DOI:** 10.3390/polym9050175

**Published:** 2017-05-14

**Authors:** Cijun Shuai, Wang Guo, Chengde Gao, Youwen Yang, Yong Xu, Long Liu, Tian Qin, Hang Sun, Sheng Yang, Pei Feng, Ping Wu

**Affiliations:** 1 State Key Laboratory of High Performance Complex Manufacturing, Central South University, Changsha 410083, China; shuai@csu.edu.cn (C.S.); guowang@csu.edu.cn (W.G.); gaochengde@csu.edu.cn (C.G.); yangyouwen@csu.edu.cn (Y.Y.); xuyong2927@csu.edu.cn (Y.X.); liulong@csu.edu.cn (L.L.); qintian@csu.edu.cn (T.Q.); shsunhang@csu.edu.cn (H.S.); 2 Key Laboratory of Organ Injury, Aging and Regenerative Medicine of Hunan Province, Changsha 410008, China; 3 Human Reproduction Center, Shenzhen Hospital of Hongkong University, Shenzhen 518000, China; yangs8@hku-szh.org; 4 College of Chemistry, Xiangtan University, Xiangtan 411105, China

**Keywords:** poly(3-hydroxybutyrate-*co*-3-hydroxyvalerate) (PHBV), calcium silicate, composite scaffolds, bioactivity, mechanical properties, cytocompatibility

## Abstract

The poor bioactivity and mechanical properties have restricted its biomedical application, although poly(3-hydroxybutyrate-*co*-3-hydroxyvalerate) (PHBV) had good biocompatibility and biodegradability. In this study, calcium silicate (CS) was incorporated into PHBV for improving its bioactivity and mechanical properties, and the porous PHBV/CS composite scaffolds were fabricated via selective laser sintering (SLS). Simulated body fluid (SBF) immersion tests indicated the composite scaffolds had good apatite-forming ability, which could be mainly attributed to the electrostatic attraction of negatively charged silanol groups derived from CS degradation to positively charged calcium ions in SBF. Moreover, the compressive properties of the composite scaffolds increased at first, and then decreased with increasing the CS content, which was ascribed to the fact that CS of a proper content could homogeneously disperse in PHBV matrix, while excessive CS would form continuous phase. The compressive strength and modulus of composite scaffolds with optimal CS content of 10 wt % were 3.55 MPa and 36.54 MPa, respectively, which were increased by 41.43% and 28.61%, respectively, as compared with PHBV scaffolds. Additionally, 3-(4,5-dimethylthiazol-2-yl)-2,5-diphenyltetrazolium bromide (MTT) assay indicated MG63 cells had a higher proliferation rate on PHBV/CS composite scaffolds than that on PHBV. Alkaline phosphatase (ALP) staining assay demonstrated the incorporation of CS significantly promoted osteogenic differentiation of MG63 cells on the scaffolds. These results suggest that the PHBV/CS composite scaffolds have the potential in serving as a substitute in bone tissue engineering.

## 1. Introduction

A scaffold, which acts as a temporary extracellular matrix, plays a significant role in regulating cell functions and tissue regeneration [1,2,3]. Therefore, it should have good biological properties, proper mechanical properties, and interconnected porous structures, as well as customized shape [4,5]. Poly (3-hydroxybutyrate-co-3-hydroxyvalerate) (PHBV) has been an attractive scaffold material due to its favorable biocompatibility, biodegradability, and processibility [6,7,8]. However, there still exist two significant problems that restrict its biomedical application, especially in bone tissue engineering. One is its lack of bioactivity, which leads to a difficulty in obtaining good integration, i.e., forming a direct contact and good interfacial bonding between the implanted scaffolds and the surrounding tissues [9]. The other is its poor mechanical properties, which is unable to provide sufficient mechanical support during tissue regeneration. Calcium silicate (CS), as a silicon-calcium based bioceramic, possesses excellent bioactivity [10,11,12] as its degradation products, including silanol groups and calcium ions, can accelerate the formation and deposition of apatite [13]. Moreover, CS can act as a rigid filler to reinforce polymer materials by microcracking and crack pinning [14,15]. In addition, it was reported that silicon ions and calcium ions released from CS could stimulate human mesenchymal stem cells proliferation and osteogenic differentiation [16,17].

Incorporating bioactive reinforcement phases into polymer scaffolds for improving bioactivity and mechanical properties has attracted much attention. Jack [18] prepared PHBV/hydroxyapatite (HA) composite scaffolds using a modified thermally induced phase-separation technique and found the introduction of HA greatly increased the stiffness and strength, and improved the in vitro bioactivity of the scaffolds. Li [15] prepared PHBV/bioglass (BG) composite scaffolds by a salt particulate leaching method and their results showed that the compressive strength and apatite-forming ability of the scaffolds were significantly improved by adding BG particles. Zhu [19] prepared silk fibroin/CS composite scaffolds by a freeze-drying method and concluded that incorporating CS into the silk fibroin can not only enhance the mechanical strength, but also improve the bioactivity and cytocompatibility of the scaffolds. However, these conventional techniques had poor control of the geometries and porous structures of scaffolds [3,20]. Selective laser sintering (SLS), an additive manufacturing technology, is quite capable of fabricating scaffolds with arbitrary geometries that matched with defected tissues. Furthermore, it is able to control the internal porous structures of scaffolds [21,22].

Based on the above consideration, in this study we incorporated CS into PHBV for the purpose of improving its bioactivity and mechanical properties, and fabricated three-dimensional porous PHBV/CS composite scaffolds via SLS in view of the potential for use as bone scaffolds. The effects of CS contents and soaking time on the apatite-forming ability of composite scaffolds were studied in simulated body fluid (SBF). Moreover, the compressive strength and modulus of composite scaffolds with different CS content were studied by compression tests. The dispersion state of CS in PHBV matrix on the surface and cross section was analyzed by scanning electron microscope (SEM). Additionally, the proliferation and osteogenic differentiation of MG63 cells cultured on PHBV/CS composite scaffolds was evaluated using MTT assay and alkaline phosphatase (ALP) staining, respectively.

## 2. Materials and Methods 

### 2.1. Materials

PHBV with poly (3-hydroxyvalerate) content of 3 mol%, molecular weight of 280 kDa, and a density of 1.25 g/cm^3^ was purchased from Tianan Biologic Material Co. (Ningbo, China). Calcium silicate (CS) powder was obtained from Kunshan Huaqiao New Materials Co. (Suzhou, China).

The following procedures [23] were mainly involved in preparing PHBV/CS composite powders: (a) ultrasonically dispersing PHBV powder of a certain amount in anhydrous ethanol for 30 min with an ultrasonic cleaner JP-040ST (Skymen Cleaning Equipment Shenzhen Co.,Ltd., Shenzhen, China); (b) adding CS powder of a proportional amount into the PHBV solution and then ultrasonically dispersing for another 30 min; (c) magnetically stirring the above PHBV/CS solution with a magnetic stirrer JB-5 (Jintan Ronghua Instrument Manufacturing Co., Ltd., Changzhou, China); (d) filtering the PHBV/CS solution to obtain the powder and then drying at 60 °C for 12 h in an electrothermal blowing dry box (101-00S, Guangzhou Daxiang Electronic Machinery Equipment Co., Ltd., Guangzhou, China); and (e) mechanically milling the dried powder using a planetary ball mill (DECO-PBM-V-0.4L, Changsha Deco Equipment Co., Ltd., Changsha, China). Five kinds of powders of different compositions were prepared by incorporating 0, 5, 10, 15, and 20 wt % CS into PHBV and labeled as PHBV, PHBV/5%CS, PHBV/10%CS, PHBV/15%CS, and PHBV/20%CS, respectively.

### 2.2. Fabrication of the Scaffold

Three-dimensional PHBV/CS composite scaffolds were fabricated using a selective laser sintering (SLS) machine, which was developed by our team. The SLS machine mainly consists of a carbon dioxide laser (SR 10i, Rofin-Sinar Laser GmbH, Hamburg, Germany) and a galvanometer-based scanning system (3D scanhead-300-15D, Beijing Century Sunny Technology Co., Beijing, China). The machine controls laser by scanning system to selectively sinter powder layers according to the cross-section profiles to form three-dimensional parts layer-by-layer. Briefly, one layer of powder was deposited on the worktable and the laser beam under the control of a computer selectively sintered the deposited powder layer according to the cross-section profiles of the designed scaffold. Then, the worktable descended by a height of a layer thickness, and subsequent powder layers were deposited and sintered directly on the top of the previously sintered layers. Afterwards, the above-mentioned layer-by-layer sintering procedure was cycled until the whole scaffold was formed. Finally, the designed scaffold was obtained after removing the unsintered powder by blowing high-pressure air. The main processing parameters of SLS were set up as follows: laser power 2 W, scanning speed 200 mm/s, scanning spacing 0.1 mm, and layer thickness 0.1 mm.

### 2.3. Microstructure Characterization

Microtopography of powders, surface, and cross section morphologies of scaffolds, and the morphology of MG63 cells cultured on scaffolds, were characterized using a scanning electron microscope (SEM) (FEI Quanta-200, FEI Co., Hillsboro, OR, USA) under high vacuum conditions at 20 kV. Element composition of the specimen surface was examined by energy-dispersive spectroscopy (EDS) (Neptune XM4, EDAX Inc., Mahwah, NJ, USA) which was installed on the SEM. All SEM specimens were oven dried, mounted on stubs, and sputtered with gold before observation. The phase composition of powders and scaffolds was identified by X-ray diffraction (XRD) (Bruker D8 Advance Diffractometer, German Bruker Co., Karlsruhe, Germany) using Cu Kα radiation (λ = 1.5406 Å) with scattering angles (2θ) range 5–70° and a scanning rate of 8°/min.

### 2.4. Compression Tests 

The compression tests of the scaffolds were carried out on a universal tester (WD-01, Shanghai Zhuoji Instruments Co., Shanghai, China) at room temperature. The loading speed was 1 mm/min. Five specimens per group were used for each composition of the scaffolds. The compressive strength and compressive modulus were determined from the compression stress-strain curve. 

### 2.5. Immersion Tests

The bioactivity of PHBV/CS composite scaffolds was assessed by immersing the scaffold specimens (with diameters of 8 mm and thicknesses of 2 mm) in simulated body fluid (SBF) which has similar ion concentrations to human blood plasma [24,25]. The SBF was changed every other day. After a predetermined incubation time, the specimens were sacrificed (three specimens per composition per time point), and then gently washed with distilled water, followed by drying at 37 °C in a electrothermal blowing dry box (101-00S). Finally, the surface morphologies and element compositions of the soaked specimens were characterized by SEM and EDS, respectively.

### 2.6. Cytocompatibility

Cell proliferation on the PHBV/CS composite scaffolds was evaluated by 3-[4,5-dimethylthiazol-2-yl]-2,5-diphenyltetrasodium bromide (MTT) assay, with the PHBV scaffolds without CS serving as the control. MG63 cells (American Type Culture Collection, Manassas, VA, USA) were cultured in low glucose Dulbecco's Modified Eagle's Medium supplemented with 10% fetal bovine serum and 1% antibiotic-antimycotic solution under 37 °C and 5% CO_2_. Before cell seeding, the scaffold specimens (with diameters of 8 mm and thicknesses of 2 mm) were sterilized by ultraviolet radiation and washed with phosphate-buffered solution (PBS), followed by being kept in the culture medium for 24 h. Then, the MG63 cells at a density of 2 × 10^5^/dm^2^ were seeded onto the scaffold specimens and cultured for different time. During the last 4 h of the predetermined culture time, the scaffold specimens were incubated with MTT. Subsequently, the precipitated formazan salts were dissolved in dimethylsulphoxide. Finally, the absorbance at 570 nm was measured by an enzyme immunosorbent assay reader.

The morphology of MG63 cells was observed by SEM. Briefly, the scaffold/cell constructs were washed with PBS and fixed with 2.5% glutaraldehyde in PBS after four days. Then, the cells on the scaffolds were dehydrated with gradient ethanol solutions, followed by drying in a drying box at 37 °C overnight. Finally, the specimens were mounted on stubs and sputtered with gold for SEM observation. Osteogenic differentiation of MG63 cells was evaluated by alkaline phosphatase (ALP) staining. Briefly, the cell/scaffold constructs were rinsed with PBS after a predetermined culture time, followed by treatment with 0.1% triton solution to obtain cell lysates. Then, the cell lysates were hydrolyzed by p-nitrophenyl phosphate in alkaline buffer solution. Finally, a light microscope was used to view the cells staining positive for ALP.

### 2.7. Statistical Analysis

The data were expressed as the mean ± standard deviation (*n* = 4). The statistical comparison was performed using Student’s t-test and differences were considered significant when *p* < 0.05. 

## 3. Results and Discussion

**Figure 1:**
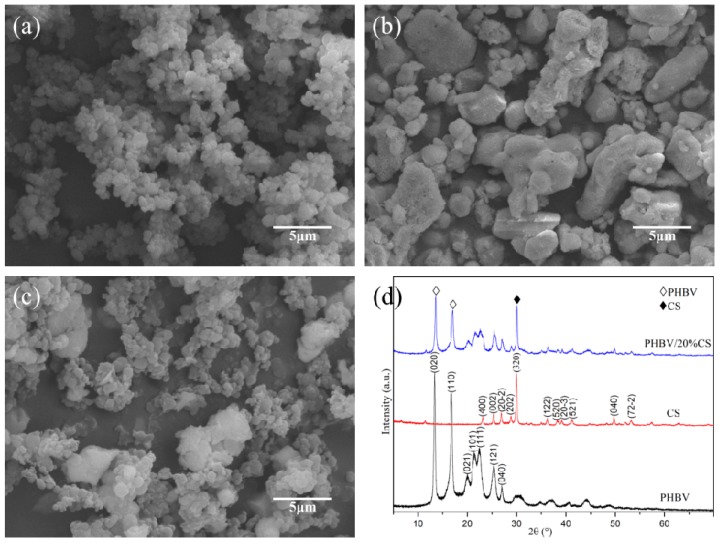
SEM (scanning electron microscope) morphologies of (**a**) PHBV; (**b**) CS; and (**c**) PHBV/20%CS composite powders and (**d**) the corresponding XRD (X-ray diffraction) patterns.

The SEM morphologies of PHBV, CS, and PHBV/20%CS powders are shown in Figure 1. The PHBV powder was spherical, with a particle size was about 1 µm (Figure 1a). The CS powder was irregular in shape and its particle size ranged mainly from 0.5 to 5 µm (Figure 1b). For the PHBV/20%CS composite powders, the CS particles were distributed randomly on the PHBV particles (Figure 1c). The corresponding XRD patterns are shown in Figure 1d. PHBV presented two strong diffraction peaks at about 2θ = 13.4 and 16.8° which were assigned to (020) and (110) planes, respectively. Additionally, other peaks at about 2θ = 20.1, 21.4, 22.6, 25.5, and 27.1° were assigned to (021), (101), (111), (121), and (040) planes, respectively [26,27]. CS presented a strong diffraction peak at about 2θ = 23.1°, which was assigned to the (400) plane. In addition, other peaks at about 2θ = 25.3, 26.9, 28.9, 30.0, 36.2, 38.2, 39.2, 41.3, 49.8, and 53.3° were assigned to (002), (20-2), (202), (320), (122), (520), (20-3), (521), (040), and (72-2) planes, respectively [28,29,30]. For the PHBV/CS composite powder, the diffraction peaks of both PHBV and CS existed. Furthermore, there existed no new phases, which indicated that no chemical reaction occurred between PHBV and CS during the preparation of the composite powders.

**Figure 2:**
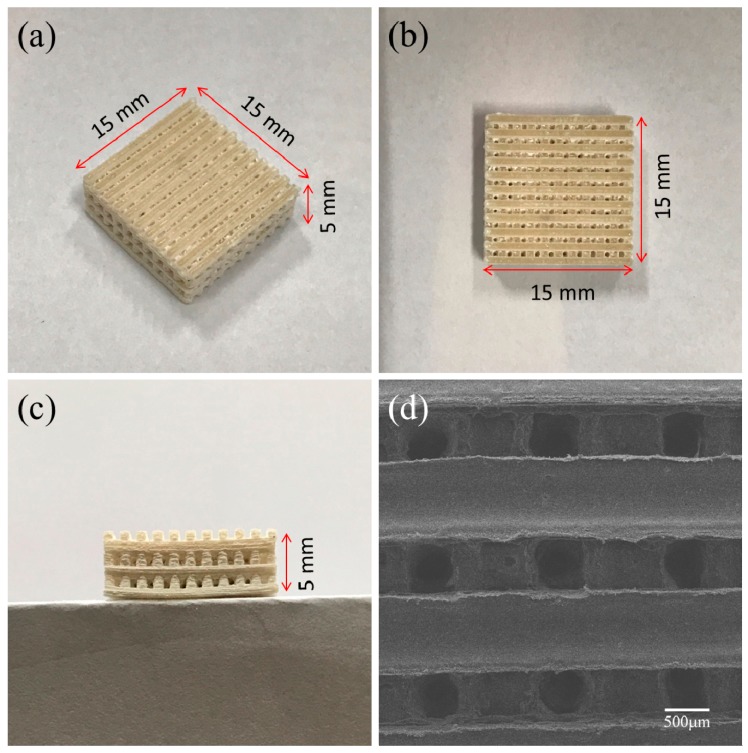
(**a**–**c**) Optical images and (**d**) SEM morphology of a representative three-dimensional rectangular PHBV/10%CS porous scaffold fabricated via SLS (selective laser sintering).

A representative three-dimensional rectangular PHBV/10%CS porous scaffold (15 mm × 15 mm × 5 mm) prepared by SLS is shown in Figure 2. It presented a well ordered and interconnected porous structure. The average pore size was approximately 500 μm (Figure 2d). This kind of interconnected porous structure may be beneficial for transport of nutrients and excretion of metabolites [31,32] and, thus, may play an important role in regulating cell functions and tissue ingrowth. Bose et al. [33] suggested that the pore size should be at least 100 μm for successful diffusion of oxygen and nutrients for cell survivability. Guo et al. [34] reported that the macropores 400–500 μm in size benefited cell infiltration and bone ingrowth. Tarafder [35] implanted tricalcium phosphate scaffolds with a 350 μm pore size into rat femurs and founded new bone formed after two weeks.

**Figure 3:**
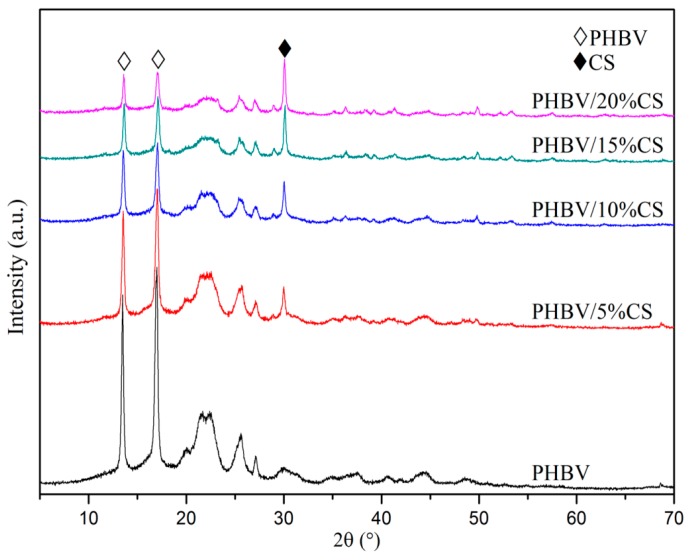
XRD patterns of the SLS-fabricated scaffolds of different formulations.

The XRD patterns of the SLS-fabricated scaffolds of different formulations were shown in Figure 3. With the increase of CS content, its diffraction peak intensity increased while PHBV decreased, which indicated that CS was blended into the composite scaffolds and did not recrystallize since its melting point (about 952 °C) [36] was far higher than that of PHBV (about 164 °C) [37]. The diffraction peak positions of PHBV and CS remained unchanged and no other peaks were detected after laser sintering, which indicated that CS was compatible with PHBV. It is noteworthy that adding only 5% CS remarkably decreased the diffraction peak of PHBV in the composite scaffold, suggesting that the incorporation of CS significantly decreased the crystallinity of PHBV. This may be due to CS particles of larger size than PHBV hindered the growth of PHBV grains during recrystallization. The crystallinity was one of the important factors that influenced the degradation rate of the polymers [38,39,40]. The decrease of crystallinity of PHBV could accelerate the degradation of the scaffolds, which may be beneficial for promoting tissue repair. 

**Figure 4:**
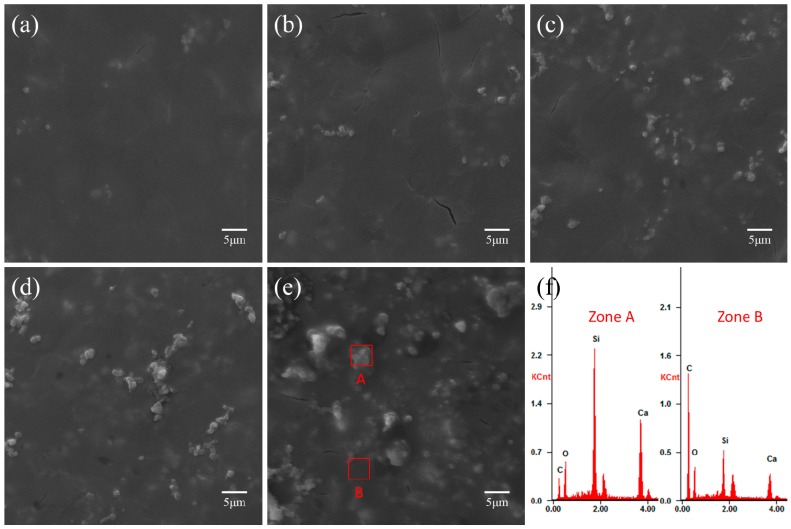
SEM surface morphologies of (**a**) PHBV scaffold; (**b**) PHBV/5%CS scaffold; (**c**) PHBV/10%CS scaffold; (**d**) PHBV/15%CS scaffold, and (**e**) PHBV/20%CS scaffold; and (**f**) the EDS (energy-dispersive spectroscopy) spectra of Zones A and B.

**Figure 5:**
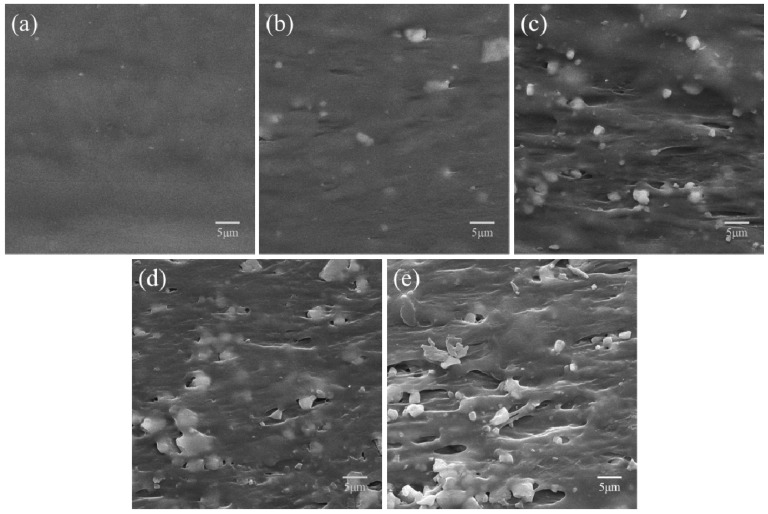
SEM cross section morphologies of (**a**) PHBV scaffold, (**b**) PHBV/5%CS scaffold, (**c**) PHBV/10%CS scaffold, (**d**) PHBV/15%CS scaffold, and (**e**) PHBV/20%CS scaffold.

The surface morphologies of PHBV and PHBV/CS composite scaffolds with different CS content were shown in Figure 4. The surface of the PHBV scaffold was flat and smooth. With the addition of 5 wt % CS, the surface became slightly rougher and several CS particles are exposed. The amount of exposed particles increases with the increase of CS, leading to a rougher surface. Figure 4f showed the EDS spectra of zones A and B. The strongest peaks corresponding to zones A and B belong to elements Si and C, respectively, which implied that they consisted mainly of CS and PHBV, respectively. Moreover, when its content is relatively low (no more than 10 wt %), the CS particles can disperse relatively uniformly in the PHBV matrix. However, the individual CS particles became continuous when its content was excessive (larger than 10 wt %). The cross section morphologies of the PHBV scaffold and PHBV/CS composite scaffolds with different CS content were shown in Figure 5. In general, the dispersion state of CS particles in the PHBV matrix on the cross-section underwent a similar change trend to that on the surface with the CS content increasing. The optimal dispersion state of CS in the PHBV matrix on the cross section also resulted from 10 wt % CS, where the particles could disperse relatively uniformly in the PHBV matrix.

**Figure 6:**
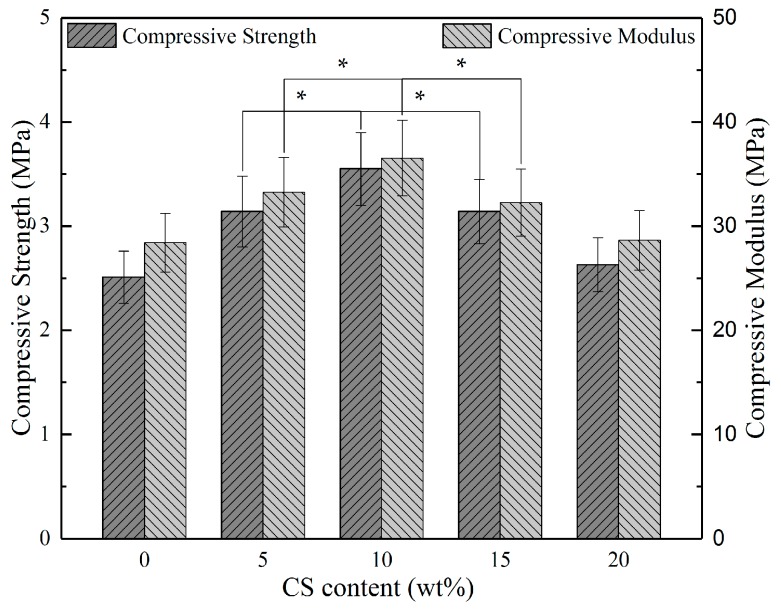
The compressive strength and compressive modulus of PHBV/CS scaffolds as a function of CS content; * represents *p* < 0.05.

Figure 6 shows the compressive strength and compressive modulus of the PHBV/CS scaffolds as a function of CS content. The compressive strength and compressive modulus of the PHBV scaffold was 2.51 MPa and 28.41 MPa, respectively. After adding 5 wt % CS, they increased by 25.10% and 17.04%, respectively. With increasing the CS content to 10 wt %, the compressive strength and modulus reached the optimal values of 3.55 MPa and 36.54 MPa, respectively, reflecting an increase of 41.43% and 28.61%, respectively. However, the compressive strength and modulus decreased when the CS content further increased to more than 10 wt %. Nevertheless, the compressive strength and modulus of all of the PHBV/CS composite scaffolds were still higher than the PHBV scaffold.

The significant increases in compressive properties of the PHBV/CS scaffolds were attributed to the strong reinforcement effects imparted by CS particles due to its high modulus and strength. Moreover, the distribution of the reinforcement phases played a significant role in influencing the compressive properties of polymer/inorganic composite scaffolds. As shown in Figure 4 and Figure 5, when the content was no more than 10%, the CS particles could disperse uniformly in the matrix so that the compressive properties increased with its content increasing. However, when the content of CS was excessive, namely more than 10%, the CS phases became continuous and, thus, decreased the compressive properties of the PHBV/CS scaffolds. It was well known that the continuous phases of stiff fillers in polymer matrixes can decrease the mechanical properties of the composites as they deteriorate the interface bonding between the fillers and matrices [41].

**Figure 7:**
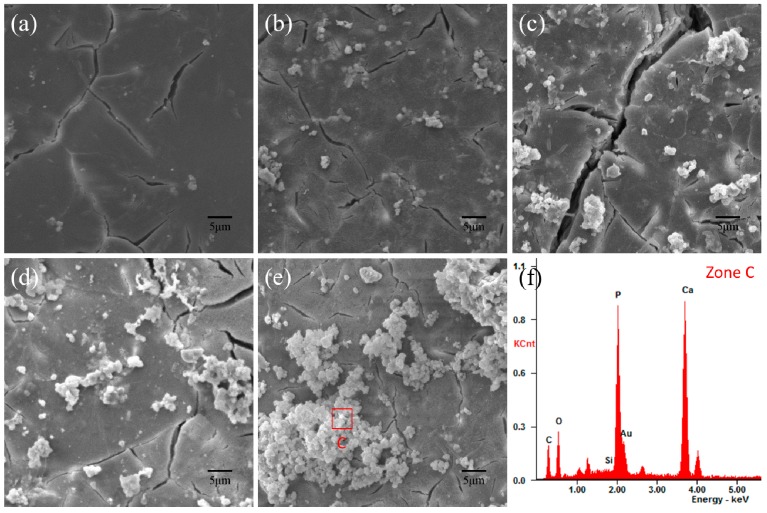
SEM surface morphologies of the (**a**) PHBV; (**b**) PHBV/5%CS; (**c**) PHBV/10%CS; (**d**) PHBV/15%CS and (**e**) PHBV/20%CS scaffolds after being immersed in SBF for 14 days; (**f**) The EDS spectrum of deposits in Zone C.

The SEM surface morphologies of the PHBV/CS scaffolds of different formulations after immersion in SBF for 14 days are shown in Figure 7. The PHBV scaffold exhibited a smooth surface (Figure 7a), whereas some particles deposited on all of the composite scaffolds. Moreover, the amount of the deposits increased with the CS content increasing. It is noticeable that large numbers of the deposits agglomerated on the surface of the PHBV/20%CS scaffold. The elemental compositions of the deposits (Zone C) were analyzed by EDS (Figure 7f). Not only were the elements Ca and O distinctly detected, but so was P. It is noteworthy that both PHBV and CS did not contain the element P, while hydroxyapatite (Ca_10_(PO_4_)_6_(OH)_2_) does. Moreover, the morphology and size of the particles in the deposits (in Figure 7) were similar to that of the hydroxyapatite deposits induced on wollastonite (CS) ceramic samples in SBF reported by Liu [42]. Therefore, it was implied that the deposits rich in P consisted mainly of hydroxyapatite.

**Figure 8:**
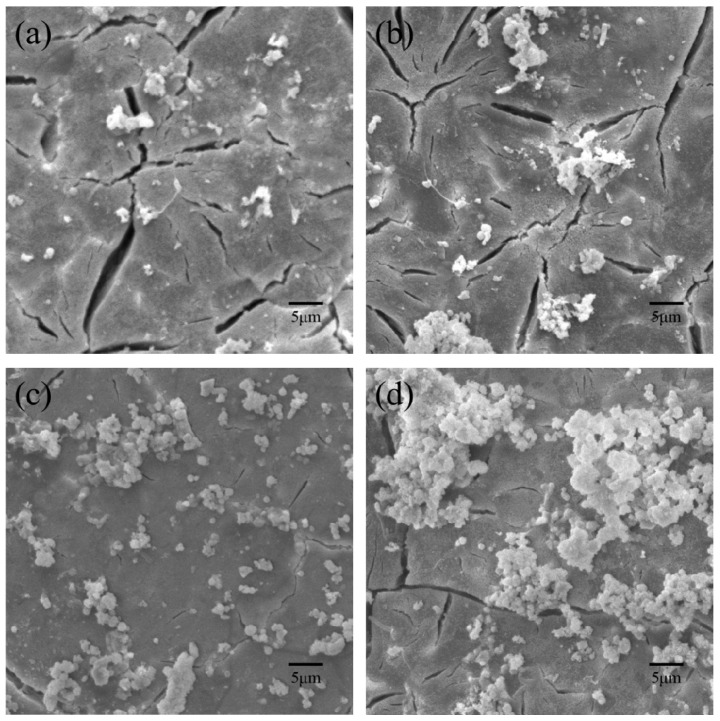
Surface morphologies of the PHBV/10%CS composite scaffolds after immersing in SBF for (**a**) 7, (**b**) 14, (**c**) 21, and (**d**) 28 days.

As the optimal content of CS was 10% for the mechanical properties, the PHBV/10%CS scaffolds were selected to further study their bioactivity for different soaking times. The surface morphologies of the PHBV/10%CS scaffolds after immersion in SBF for 7, 14, 21, and 28 days were observed using SEM (Figure 8). After seven days of immersion, there were only small amounts of hydroxyapatite depositing on the PHBV/10%CS composite scaffolds. With an increase in the immersion, the amounts of hydroxyapatite increased. After 28 days of immersion, large amounts of hydroxyapatite deposited and aggregated on the surface. The results indicated that the PHBV/10%CS composite scaffolds had favorable bioactivity.

It was well known that a negatively charged surface would absorb cations in solution [43,44,45]. The improved bioactivity of the scaffolds may be attributed to the electrostatic attraction between positively charged ions and negatively charged silanol groups derived from CS degradation. The formation processes of hydroxyapatite are briefly discussed as follows. When the PHBV/CS composite scaffold was immersed in the SBF solution, Ca^2+^ release from CS in the surface layer of the scaffold dissolved into the SBF solution. At the same time, protons from the SBF penetrated into the surface layer of the scaffold. As a result, the ion exchange (Equation (1)) occurred due to the different chemical potentials of the ions; thus, the silanol group (≡Si-OH) was created:

≡Si–O–Ca–O–Si≡ + 2H^+^ → 2≡Si-OH + Ca^2+^ (1)

With the CS continuously degrading, large amounts of silanol formed and deposited on the surface of the scaffold, and the pH of the solution increased. Subsequently, the ion exchange (Equation (2)) occurred on the surface of scaffold:

≡Si–OH + OH^−^ → ≡Si–O^−^ +H_2_O (2)

As a consequence, a negatively charged surface, which was abundant in negatively charged functional groups (≡Si–O^−^), was created. Then, the positively charged Ca^2+^ in the solution was attracted to the surface of the scaffold by the negatively charged functional group (≡Si–O^−^). Afterwards, PO_4_^2−^ was also attracted to the scaffold surface by Ca^2+^. When the ionic activity product of hydroxyapatite was high enough on the surface, the reaction, shown by Equation (3), occurred and hydroxyapatite began to nucleate:

10 Ca^2+^ + 6 PO_4_^2−^ + OH^−^ → Ca_10_(PO4)_6_(OH)_2_ (3)

The hydroxyapatite crystal nuclei spontaneously grew through continually adsorbing Ca^2+^, OH^−^, HPO_4_^−^, and PO_4_^2−^ from the SBF solution. Finally, large numbers of hydroxyapatite granules formed and aggregated on the surface of the scaffold.

**Figure 9:**
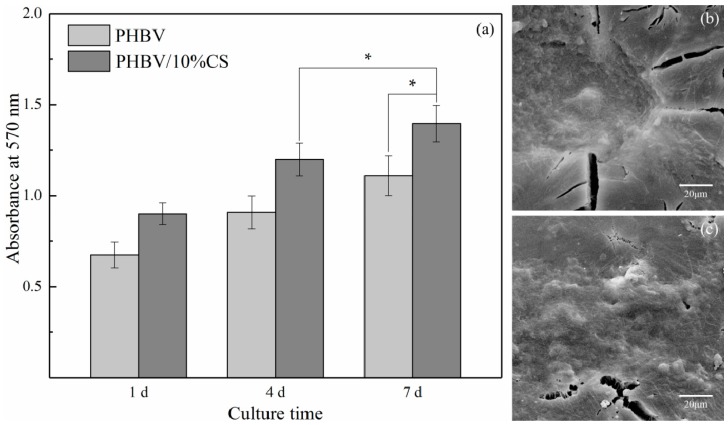
(**a**) The proliferation of MG63 cells cultured on the PHBV and PHBV/10% composite scaffolds (* represents *p* < 0.05); the SEM morphologies of MG63 cells cultured on the (**b**) PHBV and (**c**) PHBV/10% composite scaffolds on day 4.

As the PHBV/10%CS composite scaffolds had not only favorable bioactivity, but also the optimal mechanical properties, they were further studied by cell experiments, with the PHBV scaffolds serving as a control. The proliferation of MG63 cells on the scaffolds was assessed by MTT assay (Figure 9a). The absorbance values were positively correlated to the viable cell numbers. After a one-day culture, the absorbance corresponding to PHBV/10%CS scaffold was higher than that corresponding to the PHBV scaffold. As the culture time was prolonged, the proliferation level on the scaffolds both increased, which indicated MG63 cells were cytocompatible with both types of scaffolds. It is noteworthy that a remarkable difference in absorbance between PHBV/10%CS and PHBV scaffolds was observed on day 7, where the absorbance corresponding to the former was almost double that of the latter. The MTT results indicated the incorporation of CS increased the cytocompatibility of PHBV scaffolds and promoted MG63 cell proliferation. The morphologies of MG63 cells cultured on the PHBV and PHBV/10% composite scaffolds on day 4 are shown in Figure 9b,c. It could be seen that MG63 cells adhered and spread well on both of the scaffold surfaces with filopodium and lamellipodium on day 4, and there were no remarkable differences in the morphologies of MG63 cells cultured on PHBV/10%CS composite and PHBV scaffolds.

**Figure 10:**
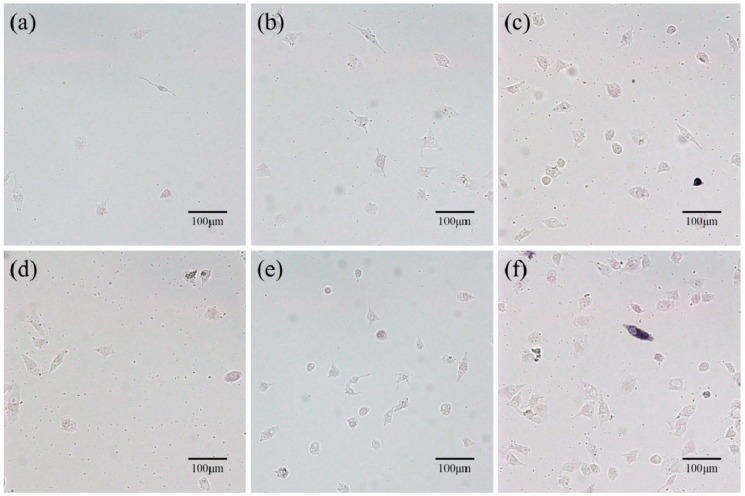
Osteogenic differentiation of MG63 cells cultured on the (**a**–**c**) PHBV scaffolds and (**d**–**f**) PHBV/10% composite scaffolds for (**a**,**d**) 1, (**b**,**e**) 4, and (**c**,**f**) 7 days. ALP (alkaline phosphatase) activity increased with the increase in culture time and the ALP activity for PHBV/10% composite scaffolds was higher than that for PHBV scaffolds at the same time point.

Osteogenic differentiation of MG63 cells cultured on the PHBV and PHBV/10% composite scaffolds was assessed by ALP staining for 1, 4, and 7 days (Figure 10). It was obvious that the ALP activity increased with increasing culture time for both types of scaffolds. More importantly, the ALP activity corresponding to PHBV/10%CS composite scaffolds was much higher than that corresponding to PHBV scaffolds at the same time point. The ALP assay results indicated that the incorporation of CS significantly improved the osteogenic differentiation of MG63 cells. 

It was known that the surface characteristics of a material can influence the cell response and behavior [46]. Postiglione [47] reported that bone tissue showed better interactions with titanium implants of a rough surface compared with a relatively smooth one. Deligianni et al. [48] founded the cell adhesion, proliferation, differentiation, and detachment strength of human bone marrow cells on hydroxyapatite discs increased as the roughness of the discs increased. As shown in Figure 4, the incorporation of CS changed the flat and smooth surface morphology of the PHBV scaffold to a relatively rough surface. Such a rough surface with exposed CS particles may provide more target spots for cell adhesion and thereby facilitate cell proliferation. Furthermore, the cell-material interactions were also influenced by the ion species and level released from the material. Xynos et al. [49] cultured osteoblasts on bioactive glass and found that the ion products released from the glass, especially Si and Ca, could stimulate cell proliferation. Shie et al. [50] found that the silicon ions of an appropriate concentration could effectively support the proliferation of osteoblast-like cells and positively stimulate biological responses in MG63 cells through producing bone-specific proteins. Moreover, Ca ions could activate Ca-sensing receptors in osteoblast cells, thus increasing the expression of growth factors, e.g., IGF-I or IGF-II [51,52], and favored osteoblast proliferation, differentiation, and extracellular matrix (ECM) mineralization [53]. Furthermore, Si ions were reported to likely be the key factor for mediating the mineralization and nodule formation [54]. Therefore, it was expected that the Si and Ca ions released from the CS component in the composite scaffolds may play a significant role in promoting MG63 cell proliferation and osteogenic differentiation.

## 4. Conclusions

Three-dimensional PHBV/CS composite scaffolds were fabricated via SLS and exhibited a well ordered and interconnected porous structure. The incorporation of CS remarkably improved the bioactivity of the scaffolds, which could be attributed to the fact that the degradation products of CS including silanol groups and calcium ions could accelerate the formation and deposition of apatite through electrostatic attraction. Moreover, the compressive strength and modulus were increased by introducing CS, and the enhancement efficiency was dependent on the amount and dispersion state of CS particles in the PHBV matrix. The optimal CS content was 10 wt %, where the compressive strength and compressive modulus were 3.55 and 36.54 MPa, respectively, which was an increase of 41.43% and 28.61%, respectively, compared with the PHBV scaffolds. Additionally, the incorporation of CS significantly promoted the proliferation and osteogenic differentiation of MG63 cells on the scaffolds. This study indicated the biological and mechanical enhanced PHBV scaffolds by incorporating CS may be a promising substitution for bone tissue engineering. 

## References

[1] Hollister S.J. (2005). Porous scaffold design for tissue engineering. Nat. Mater..

[2] Hutmacher D.W. (2001). Scaffold design and fabrication technologies for engineering tissues—State of the art and future perspectives. J. Biomat. Sci-Polym. Ed..

[3] Yeong W.-Y., Chua C.-K., Leong K.-F., Chandrasekaran M. (2004). Rapid prototyping in tissue engineering: Challenges and potential. Trends Biotechnol..

[4] Raghunath J., Rollo J., Sales K.M., Butler P.E., Seifalian A.M. (2007). Biomaterials and scaffold design: Key to tissue-engineering cartilage. Biotechnol. Appl. Biochem..

[5] Burg K.J., Porter S., Kellam J.F. (2000). Biomaterial developments for bone tissue engineering. Biomaterials.

[6] Chen G.Q., Wu Q. (2005). The application of polyhydroxyalkanoates as tissue engineering materials. Biomaterials.

[7] Zhao K., Deng Y., Chen J.C., Chen G.-Q. (2003). Polyhydroxyalkanoate (PHA) scaffolds with good mechanical properties and biocompatibility. Biomaterials.

[8] Sabir M.I., Xu X., Li L. (2009). A review on biodegradable polymeric materials for bone tissue engineering applications. J. Mater. Sci..

[9] Mistry A.S., Pham Q.P., Schouten C., Yeh T., Christenson E.M., Mikos A.G., Jansen J.A. (2010). In vivo bone biocompatibility and degradation of porous fumarate-based polymer/alumoxane nanocomposites for bone tissue engineering. J. Biomed. Mater. Res. A.

[10] Xu S., Lin K., Wang Z., Chang J., Wang L., Lu J., Ning C. (2008). Reconstruction of calvarial defect of rabbits using porous calcium silicate bioactive ceramics. Biomaterials.

[11] Xue W., Liu X., Zheng X., Ding C. (2005). In vivo evaluation of plasma-sprayed wollastonite coating. Biomaterials.

[12] Liu X., Morra M., Carpi A., Li B. (2008). Bioactive calcium silicate ceramics and coatings. Biomed. Pharmacother..

[13] Liu X., Ding C., Chu P.K. (2004). Mechanism of apatite formation on wollastonite coatings in simulated body fluids. Biomaterials.

[14] Unal H., Mimaroglu A., Alkan M. (2003). Mechanical properties and morphology of nylon-6 hybrid composites. Polym. Int..

[15] Li H., Du R., Chang J. (2005). Fabrication, characterization, and in vitro degradation of composite scaffolds based on PHBV and bioactive glass. J. Biomater. Appl..

[16] Fei L., Wang C., Xue Y., Lin K., Chang J., Sun J. (2012). Osteogenic differentiation of osteoblasts induced by calcium silicate and calcium silicate/β-tricalcium phosphate composite bioceramics. J. Biomed. Mater. Res. B.

[17] Saravanan S., Vimalraj S., Vairamani M., Selvamurugan N. (2015). Role of mesoporous wollastonite (calcium silicate) in mesenchymal stem cell proliferation and osteoblast differentiation: A cellular and molecular study. J. Biomed. Nanotechnol..

[18] Jack K.S., Velayudhan S., Luckman P., Trau M., Grøndahl L., Cooper-White J. (2009). The fabrication and characterization of biodegradable HA/PHBV nanoparticle–polymer composite scaffolds. Acta Biomater..

[19] Zhu H., Shen J., Feng X., Zhang H., Guo Y., Chen J. (2010). Fabrication and characterization of bioactive silk fibroin/wollastonite composite scaffolds. Mat. Sci. Eng. C.

[20] Gao C., Deng Y., Feng P., Mao Z., Li P., Yang B., Deng J., Cao Y., Shuai C., Peng S. (2014). Current progress in bioactive ceramic scaffolds for bone repair and regeneration. Int. J. Mol. Sci..

[21] Williams J.M., Adewunmi A., Schek R.M., Flanagan C.L., Krebsbach P.H., Feinberg S.E., Hollister S.J., Das S. (2005). Bone tissue engineering using polycaprolactone scaffolds fabricated via selective laser sintering. Biomaterials.

[22] Tan K., Chua C., Leong K., Cheah C., Cheang P., Bakar M.A., Cha S. (2003). Scaffold development using selective laser sintering of polyetheretherketone–hydroxyapatite biocomposite blends. Biomaterials.

[23] Pei F., Peng S., Ping W., Gao C., Wei H., Deng Y., Shuai C. (2016). A space network structure constructed by tetraneedlelike ZnO whiskers supporting boron nitride nanosheets to enhance comprehensive properties of poly(l-lacti acid) scaffolds. Sci. Rep..

[24] Kokubo T., Takadama H. (2006). How useful is SBF in predicting in vivo bone bioactivity?. Biomaterials.

[25] Shuai C., Feng P., Wu P., Liu Y., Liu X., Lai D., Gao C., Peng S. (2017). A combined nanostructure constructed by graphene and boron nitride nanotubes reinforces ceramic scaffolds. Chem. Eng. J..

[26] Lei C., Zhu H., Li J., Li J., Feng X., Chen J. (2014). Preparation and characterization of polyhydroxybutyrate- co -hydroxyvalerate/silk fibroin nanofibrous scaffolds for skin tissue engineering. Polym. Eng. Sci..

[27] Ten E., Jiang L., Wolcott M.P. (2012). Crystallization kinetics of poly(3-hydroxybutyrate-co-3-hydroxyvalerate)/cellulose nanowhiskers composites. Carbohyd. Polym..

[28] Pei L.Z., Yang L.J., Yang Y., Fan C.G., Yin W.Y., Chen J., Zhang Q.F. (2010). A green and facile route to synthesize calcium silicate nanowires. Mater. Charact..

[29] Tiimob B.J., Rangari V.K., Jeelani S. (2014). Effect of reinforcement of sustainable β-casio3 nanoparticles in bio-based epoxy resin system. J. Appl. Polym. Sci..

[30] Gao C., Liu T., Shuai C., Peng S. (2014). Enhancement mechanisms of graphene in nano-58S bioactive glass scaffold: Mechanical and biological performance. Sci. Rep..

[31] Feng P., Niu M., Gao C., Peng S., Shuai C. (2014). A novel two-step sintering for nano-hydroxyapatite scaffolds for bone tissue engineering. Sci. Rep..

[32] Zhang N., Wang Y., Xu W., Hu Y., Ding J. (2016). Poly(lactide-co-glycolide)/hydroxyapatite porous scaffold with microchannels for bone regeneration. Polymers.

[33] Bose S., Roy M., Bandyopadhyay A. (2012). Recent advances in bone tissue engineering scaffolds. Trends Biotechnol..

[34] Guo H., Su J., Wei J., Kong H., Liu C. (2009). Biocompatibility and osteogenicity of degradable Ca-deficient hydroxyapatite scaffolds from calcium phosphate cement for bone tissue engineering. Acta Biomater..

[35] Tarafder S., Balla V.K., Davies N.M., Bandyopadhyay A., Bose S. (2013). Microwave sintered 3D printed tricalcium phosphate scaffolds for bone tissue engineering. J. Tissue Eng. Regen. M..

[36] Cai Y.Q., Lin Y.Y., Li X., Huang J.T., Aoki T. (2012). In Calcium behaviors in MnZn ferrite at different temperatures. Key Eng. Mat..

[37] Jenkins M., Cao Y., Howell L., Leeke G. (2007). Miscibility in blends of poly(3-hydroxybutyrate-co-3-hydroxyvalerate) and poly(ɛ-caprolactone) induced by melt blending in the presence of supercritical CO_2_. Polymer.

[38] Tsuji H., Ikada Y. (2000). Properties and morphology of poly(l-lactide) 4. Effects of structural parameters on long-term hydrolysis of poly(l-lactide) in phosphate-buffered solution. Polym. Degrad. Stabil..

[39] Loo S.C., Ooi C.P., Wee S.H., Boey Y.C. (2005). Effect of isothermal annealing on the hydrolytic degradation rate of poly(lactide-co-glycolide) (PLGA). Biomaterials.

[40] Hurrell S., Cameron R.E. (2002). The effect of initial polymer morphology on the degradation and drug release from polyglycolide. Biomaterials.

[41] Fu S.-Y., Feng X.-Q., Lauke B., Mai Y.-W. (2008). Effects of particle size, particle/matrix interface adhesion and particle loading on mechanical properties of particulate–polymer composites. Compos. Part B-Eng..

[42] Liu X., Ding C., Wang Z. (2001). Apatite formed on the surface of plasma-sprayed wollastonite coating immersed in simulated body fluid. Biomaterials.

[43] Ohgaki M., Kizuki T., Katsura M., Yamashita K. (2001). Manipulation of selective cell adhesion and growth by surface charges of electrically polarized hydroxyapatite. J. Biomed. Mater. Res..

[44] Kabaso D., Gongadze E., Perutková S., Matschegewski C., Kralj-Iglic V., Beck U., Van R.U., Iglic A. (2011). Mechanics and electrostatics of the interactions between osteoblasts and titanium surface. Comput. Method Biomech..

[45] Liu X., Zhao X., Fu R.K., Ho J.P., Ding C., Chu P.K. (2005). Plasma-treated nanostructured TiO_2_ surface supporting biomimetic growth of apatite. Biomaterials.

[46] Anselme K. (2000). Osteoblast adhesion on biomaterials. Biomaterials.

[47] Postiglione L., Di Domenico G., Ramaglia L., Di Lauro A., Di Meglio F., Montagnani S. (2004). Different titanium surfaces modulate the bone phenotype of SaOS-2 osteoblast-like cells. Eur. J. Histochem..

[48] Deligianni D.D., Katsala N.D., Koutsoukos P.G., Missirlis Y.F. (2001). Effect of surface roughness of hydroxyapatite on human bone marrow cell adhesion, proliferation, differentiation and detachment strength. Biomaterials.

[49] Xynos I.D., Edgar A.J., Buttery L.D., Hench L.L., Polak J.M. (2000). Ionic products of bioactive glass dissolution increase proliferation of human osteoblasts and induce insulin-like growth factor ii mRNA expression and protein synthesis. Biochem. Bioph. Res. Commun..

[50] Shie M.Y., Ding S.J., Chang H.C. (2011). The role of silicon in osteoblast-like cell proliferation and apoptosis. Acta Biomater..

[51] Marie P.J. (2010). The calcium-sensing receptor in bone cells: A potential therapeutic target in osteoporosis. Bone.

[52] Valerio P., Pereira M., Goes A., Leite M.F. (2009). Effects of extracellular calcium concentration on the glutamate release by bioactive glass (BG60S) preincubated osteoblasts. Biomed Mater.

[53] Maeno S., Niki Y., Matsumoto H., Morioka H., Yatabe T., Funayama A., Toyama Y., Taguchi T., Tanaka J. (2005). The effect of calcium ion concentration on osteoblast viability, proliferation and differentiation in monolayer and 3D culture. Biomaterials.

[54] Gough J.E., Jones J.R., Hench L.L. (2004). Nodule formation and mineralisation of human primary osteoblasts cultured on a porous bioactive glass scaffold. Biomaterials.

